# Supplementary material for: Antibiotic Resistance and Mobile Genetic Elements in Extensively Drug-Resistant Klebsiella pneumoniae Sequence Type 147 Recovered from Germany
Source: Antibiotics (Basel). 2020 Oct 5;9(10):675. doi: 10.3390/antibiotics9100675 (PMC7600919; doi:10.3390/antibiotics9100675)
Supplement: Supplementary file 1 [file antibiotics-09-00675-s001.pdf]

Supplementary Materials

Table S1. List of oligonucleotides used in the present study.

Target	Primer	Sequence (5′ to 3′)	Experiment	Reference
IncR	L7	TCGCTTCATTCCTGCTTCAGC	Southern blot	This study
	L8	GTGTGCTGTGGTTATGCCTCA		
StrA	L3	CTGTCAGAGGCGGAGAATCT	Southern blot	This study
	L4	ATCCGCTCCAGACAGATCAG		
RpoB	KX65	TGGTAAACGTCCACAAGTTCTG	Southern blot	This study
	KX66	CTGTAGCTCTGAATCGGGAAT		
Terminase	KX103	TTCAAGCTCTTTCATCGCCG	Southern blot	This study
	KX104	TTATGAAATGGGCGTGCTGG		
pHKP0018.1	KX37	CGGTAATGTTAGGGGCTGGA	PCR-based gap closure	This study
	KX38	CACTGACAATGGTGCCACTC		
pHKP0018.3	KX45	GATCTGGTGGCGGACTATCA	PCR-based gap closure	This study
	KX46	TCAGGAACGTTTAAAGGCGC		
pHKP0018.3	KX47	CCGGACAAGTATTTCTGCGG	PCR-based gap closure	This study
	KX48	CTGCTTTGTCGTCCGGTAAG		
pHKP0018.4	KX61	CCACGTTCAACAGCTCCATC	PCR-based gap closure	This study
	KX62	CTGACCTCCTGAAACGCTCT		

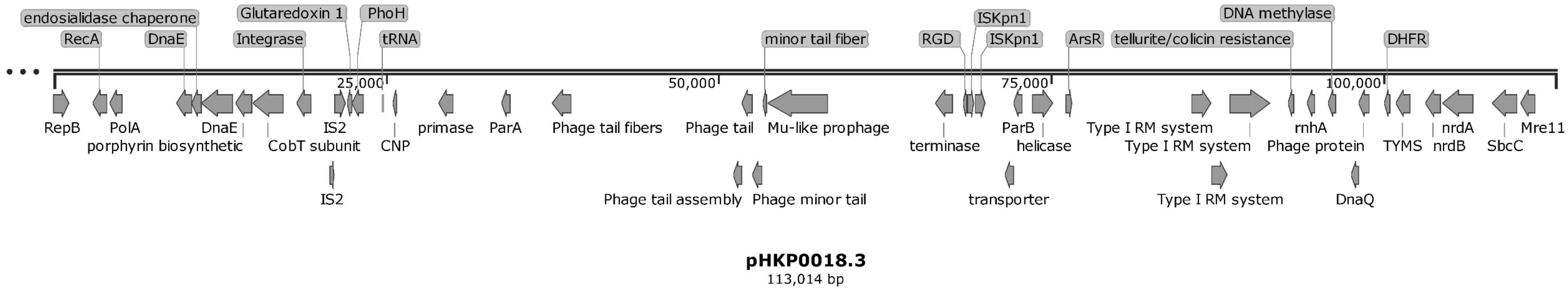

Figure S1. Major structural features of the IncFIB plasmid pHKP0018.3 identified in *K. pneumoniae* isolates HKP0018, HKP0064 and HKP0067. Arrows represent predicted ORFs and their direction represents the direction of transcription. Hypothetical proteins are not shown.

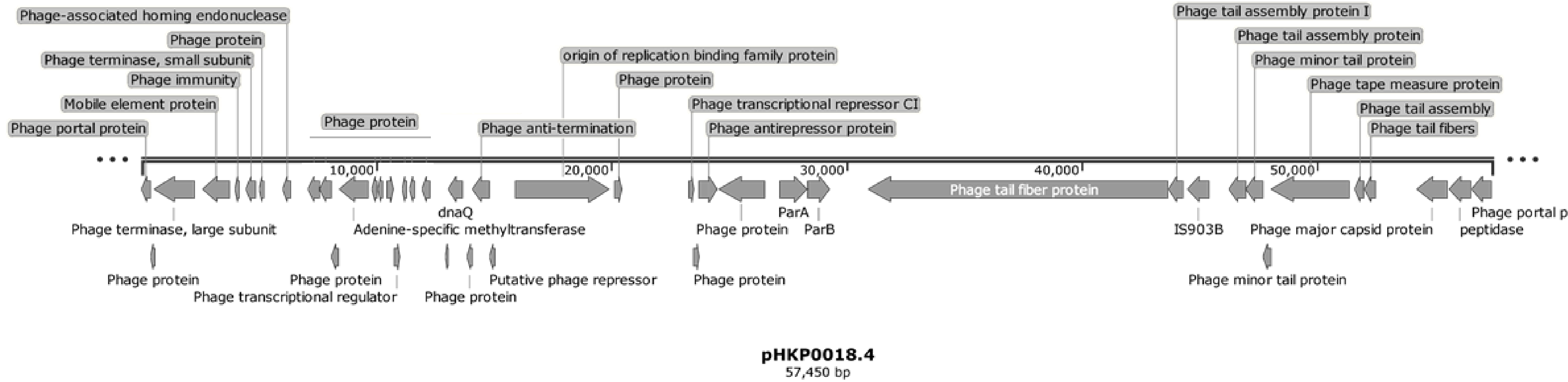

Figure S2. Major structural features of the pHKP0018.4 plasmid identified in *K. pneumoniae* isolates HKP0018, HKP0064 and HKP0067. Arrows represent predicted ORFs and their direction represents the direction of transcription. Hypothetical proteins are not shown.

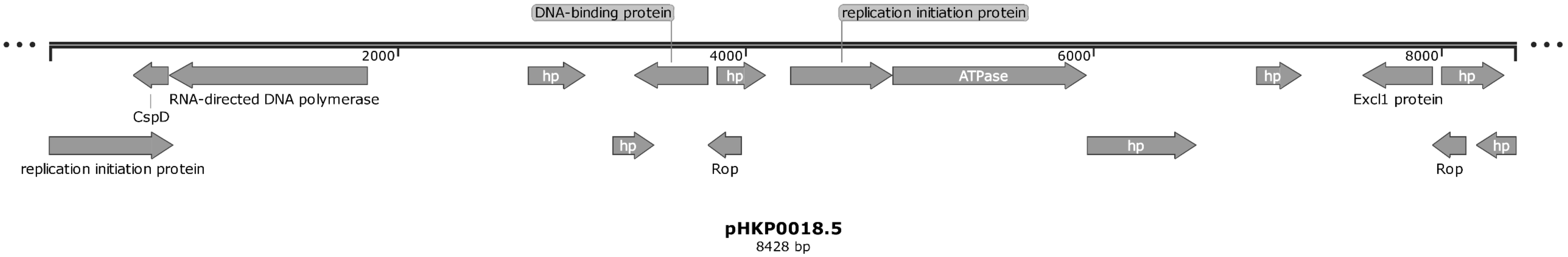

Figure S3. Major structural features of pHKP0018.5 identified in *K. pneumoniae* isolates HKP0018, HKP0064 and HKP0067. Arrows represent predicted ORFs and their direction represents the direction of transcription. Hypothetical proteins are assigned as hp.

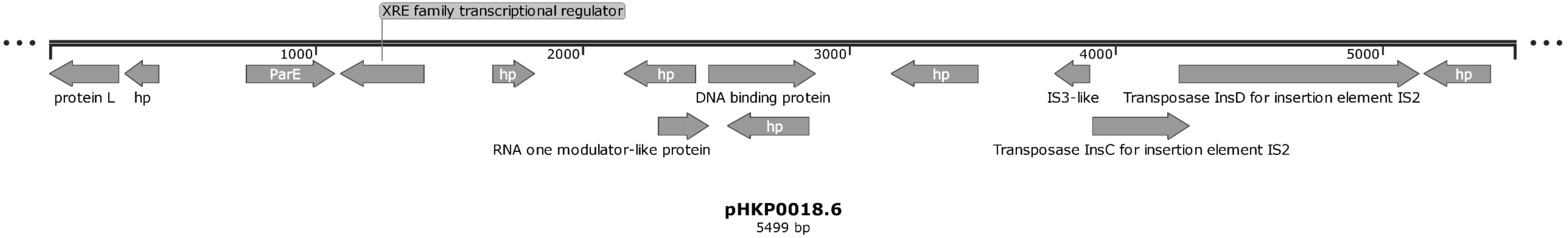

Figure S4. Major structural features of pHKP0018.6 identified in *K. pneumoniae* isolates HKP0018, HKP0064 and HKP0067, respectively. Arrows represent predicted ORFs and the direction of the arrow represents the direction of transcription. Hypothetical proteins are assigned as hp.
